# Temporal changes in immune cell composition and cytokines in response to chemoradiation in rectal cancer

**DOI:** 10.1038/s41598-018-25970-z

**Published:** 2018-05-15

**Authors:** Yong Joon Lee, Sat Byol Lee, Suk Kyung Beak, Yoon Dae Han, Min Soo Cho, Hyuk Hur, Kang Young Lee, Nam Kyu Kim, Byung Soh Min

**Affiliations:** 1 Graduate School of Medical Science and Engineering, Korea Advanced Institute of Science and Technology, Daejeon, Republic of Korea; 2 Open NBI Convergence Technology Laboratory, Avison Biomedical Research Centre, Yonsei University College of Medicine, Seoul, Republic of Korea; 3 Department of Surgery, Severance Hospital, Yonsei University College of Medicine, Seoul, Republic of Korea

## Abstract

We measured systemic changes in the immune response in 92 patients receiving preoperative chemoradiation therapy (CRT) and subsequent surgery for rectal cancer. The peripheral blood was sampled five times from the onset of CRT until surgery. Lymphocytes decreased continuously during CRT but increased after CRT. The increased lymphocyte population was predominantly CD8+ T lymphocytes, which accounted for a significantly larger proportion in patients without residual lymph node metastasis than in those with residual lymph node metastasis. Neutrophils and monocytes decreased during the initial two weeks of CRT but were maintained or increased afterwards. Neutrophil and monocyte counts were significantly lower in patients with a pCR (pathologic complete response) than in those without a pCR two weeks after CRT began but not at the initiation of CRT. All cytokines showed dramatic changes one month after the termination of CRT. Cytokines related to the antitumour immune response increased, and those related to tumour progression decreased. The predictive value of cytokines was not clear. In short, we observed that immune components in peripheral blood are affected by CRT and show dynamic changes over time. We identified biomarker candidates to predict the pathologic response in the future.

## Introduction

Chemoradiation therapy (CRT) is one of several effective options available for the treatment of solid tumours under various clinical settings. It is well known that CRT causes tumour regression through direct cytotoxicity and cytostaticity. However, accumulating evidence implies that CRT can also induce an antitumour immune response^1–3^. The immunomodulatory potential of radiotherapy was initially demonstrated through interpretation of the abscopal effect, which refers to the phenomenon of tumours shrinking outside the scope of localized radiotherapy. Thereafter, therapeutic approaches have focussed on harnessing the antitumour immune response by adjusting clinical radiation protocols. Additionally, it is well known that some chemotherapeutic agents can trigger the antitumour immune response by eliciting immunologic cell death^4^.

To date, several immunologic molecules associated with the therapeutic mechanisms of CRT have been suggested as biomarkers that forecast therapeutic success or failure^5–8^. However, previous studies have questioned the reliability and reproducibility of these suggested biomarkers. Additionally, much remains to be learned about how an effective antitumour immune response is induced by chemoradiation. Hopefully, the recent emergence of immune checkpoint inhibitors and proven clinical effects to support their use in the treatment of various cancers will make it possible to enhance the efficacy of CRT by properly combining it with immunotherapy^9,10^.

In locally advanced rectal cancer (LARC), neoadjuvant CRT followed by radical surgery reduces locoregional recurrence and improves long-term survival^11^. Based on these advantages, CRT has become the standard therapeutic modality for patients with LARC. However, the tumour response to CRT varies among patients: only approximately 20% of patients show a pathologic complete response (pCR), approximately 60% show a partial response, and the remaining 20% do not respond to treatment^12^. Continuous efforts have been undertaken to identify a highly predictive biomarker in order to provide other favourable therapeutic options for patients who are unresponsive to treatment. Additionally, we need to better understand the immunologic mechanism of chemoradiation to establish an optimal immunotherapeutic strategy.

Thus, we decided to explore the chronological changes in the systemic immune response during and after CRT in LARC patients. We measured the changes in immune cell composition and cytokines in peripheral blood that reflect the systemic immune response. We aimed to provide a comprehensive understanding of the immunologic mechanism of chemoradiation and to investigate potential candidate biomarkers as reliable predictors of the pathologic treatment response.

## Results

### Characteristics of patients and treatment schedule

**Figure 1:**
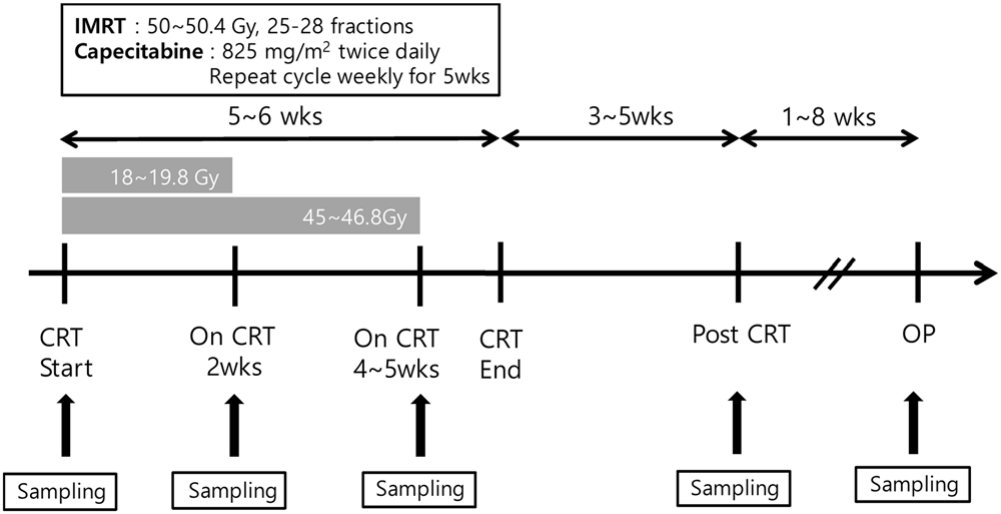
Schematic diagram of treatment schedule and peripheral blood sampling schedule. IMRT = intensity-modulated radiation therapy; Gy = greys; wks = weeks; CRT = chemoradiation therapy; OP = operation.

The clinicopathologic characteristics of the patients are shown in Table 1. All patients received preoperative oral capecitabine-based chemotherapy and fractionated intensity-modulated radiotherapy (IMRT). The cumulative radiation dose at each time point, treatment intervals, and blood sampling schedules are presented in Fig. 1. Radical surgeries were performed between four and twelve weeks after the termination of preoperative CRT. Approximately 20% of patients showed a pCR, Mandard grade 1, and almost 70% of patients were confirmed to have no residual lymph node metastasis upon pathological examination of surgical specimens.

**Table 1:** Clinicopathological characteristics.

N = 92	
**Age**	57.35 ± 11.01
**Sex**	
Male	66 (71.7%)
Female	26 (28.3%)
**Histopathology**	
AWD/AMD	87 (94.6%)
APD	2 (2.2%)
Mucinous	3 (3.2%)
**Clinical T Stage**	
cT2	7 (7.6%)
cT3	75 (81.5%)
cT4	10 (10.9%)
**Clinical N Stage**	
negative	22 (23.9%)
positive	70 (76.1%)
**CRM invasion**	
negative	27 (29.3%)
positive	65 (70.7%)
**Mandard grade**	
1 (pCR)	19 (20.7%)
2	17 (18.5%)
3	44 (47.8%)
4	12 (13.0%)
**ypN stage**	
yN0	63 (68.5%)
yN1	23 (25.0%)
yN2	6 (6.5%)

Values are presented as the mean ± standard deviation or number (percentage). AWD = well-differentiated adenocarcinoma; AMD = moderately differentiated adenocarcinoma; APD = poorly differentiated adenocarcinoma; CRM = circumferential resection margin.

### Complete blood count (CBC) analysis and leukocyte composition

**Figure 2:**
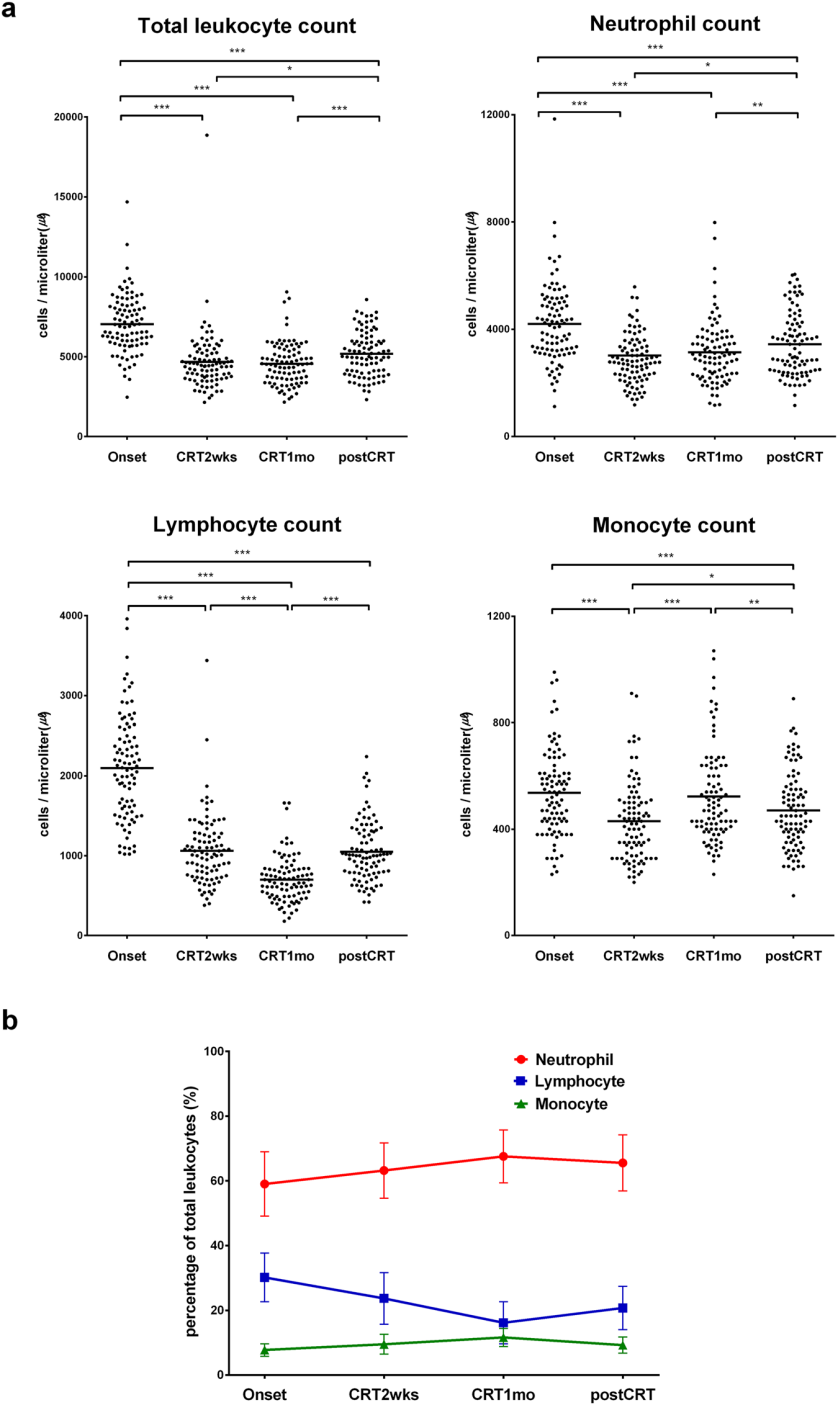
Changes in leukocyte composition in response to CRT: complete blood count (CBC) analysis. (**a**) Absolute numbers of total leukocytes, neutrophils, lymphocytes, and monocytes in patients (n = 92) during and after CRT are indicated by dots. Repeated measures analysis of variance (ANOVA) demonstrated statistically significant effects of time; total leukocytes p < 0.001; neutrophils p < 0.001; lymphocytes p < 0.001; and monocytes p < 0.001. Two-tailed paired Student’s t-test was performed to analyse differences between time points. *p < 0.05; **p < 0.0083 (adjusted significance level by Bonferroni’s method); ***p < 0.001. (**b**) Changes in the ratios of neutrophils, monocytes, and lymphocytes to total leukocytes (percentage of total leukocytes). Data are presented as the means ± standard deviations (SDs). Onset = the onset of CRT; CRT2wks = 2 weeks after the onset of CRT; CRT1mo = 1 month after the onset of CRT; postCRT = 1 month after the termination of CRT.

**Figure 3:**
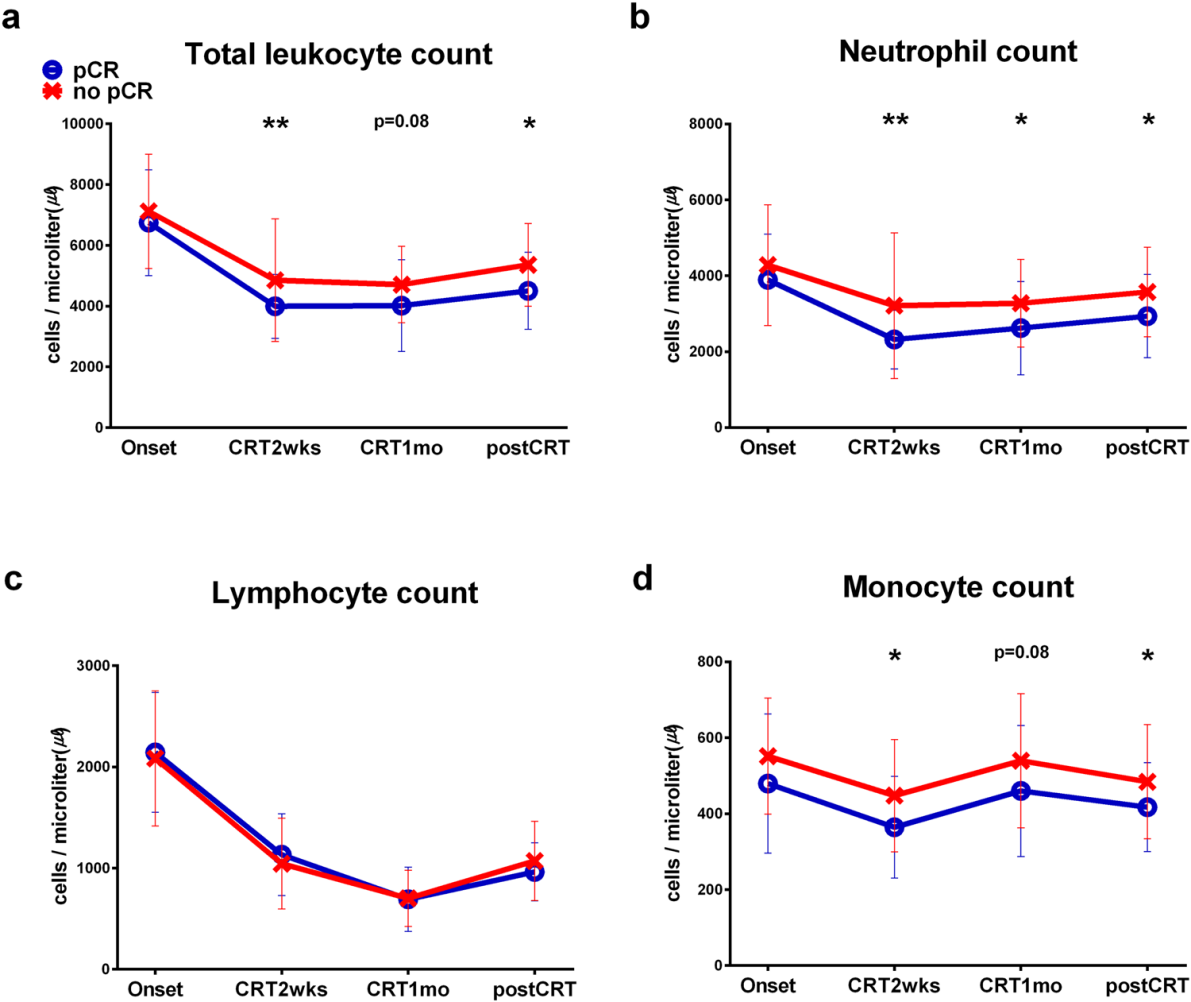
Predictive value of leukocyte components regarding pathologic complete response (pCR). Patients were divided into two groups: patients with a pCR (n = 19) and patients without a pCR (n = 73). (**a**) Mean (±standard deviation, SD) total leukocyte counts of the two groups at each time point. Repeated measures ANOVA; time effect p < 0.001; group effect p = 0.031; time-group interaction p = 0.616. (**b**) Mean (±SD) neutrophil counts of the two groups at each time point. Repeated measures ANOVA; time effect p < 0.001; group effect p = 0.020; time-group interaction p = 0.610. (**c**) Mean (±SD) lymphocyte counts of the two groups at each time point. Repeated measures ANOVA; time effect p < 0.001; group effect p = 0.916; time-group interaction p = 0.343. (**d**) Mean (±SD) monocyte counts of the two groups at each time point. Repeated measures ANOVA; time effect p < 0.001; group effect p = 0.012; time-group interaction p = 0.983. The intergroup differences were analysed using the two-tailed Welch’s t-test. *p < 0.05; **p < 0.0125 (adjusted significance level by Bonferroni’s method); ***p < 0.001. Onset = the onset of CRT; CRT2wks = 2 weeks after the onset of CRT; CRT1mo = 1 month after the onset of CRT; postCRT = 1 month after the termination of CRT.

During the initial two weeks of CRT, total leukocyte, neutrophil, lymphocyte, and monocyte counts decreased (Fig. 2a). At one month after the onset of CRT, lymphocyte count further decreased as cumulative chemoradiation dose increased, but monocyte count increased; total leukocyte count and neutrophil count showed no significant changes. One month after the termination of CRT, total leukocyte count, neutrophil count and lymphocyte count, but not monocyte count, were increased. The change in leukocyte composition during and after CRT revealed that the lymphocyte to leukocyte ratio decreased during the course of CRT and began to recover after CRT was terminated (Fig. 3b). On the other hand, the neutrophil to leukocyte ratio and the monocyte to leukocyte ratio increased during CRT and decreased after CRT.

### Predictive value of changes in leukocyte composition

We investigated the relationship between changes in leukocyte composition and the therapeutic response to CRT. There were no significant differences in lymphocyte count at any time point between patients with and without a pCR. At the onset of CRT, total leukocyte, neutrophil, and monocyte counts were not significantly different between patients with and without a pCR. However, two weeks after initiating CRT, patients with a pCR had significantly lower total leukocyte, neutrophil, and monocyte counts than those without a pCR, and this trend continued (Fig. 3). Additionally, monocyte count significantly differed between patients with and without residual lymph node metastasis at two weeks after the onset of CRT, but there were no differences in the other cell counts (Supplementary Fig. S2).

### Changes in T lymphocyte composition and their predictive value

**Figure 4:**
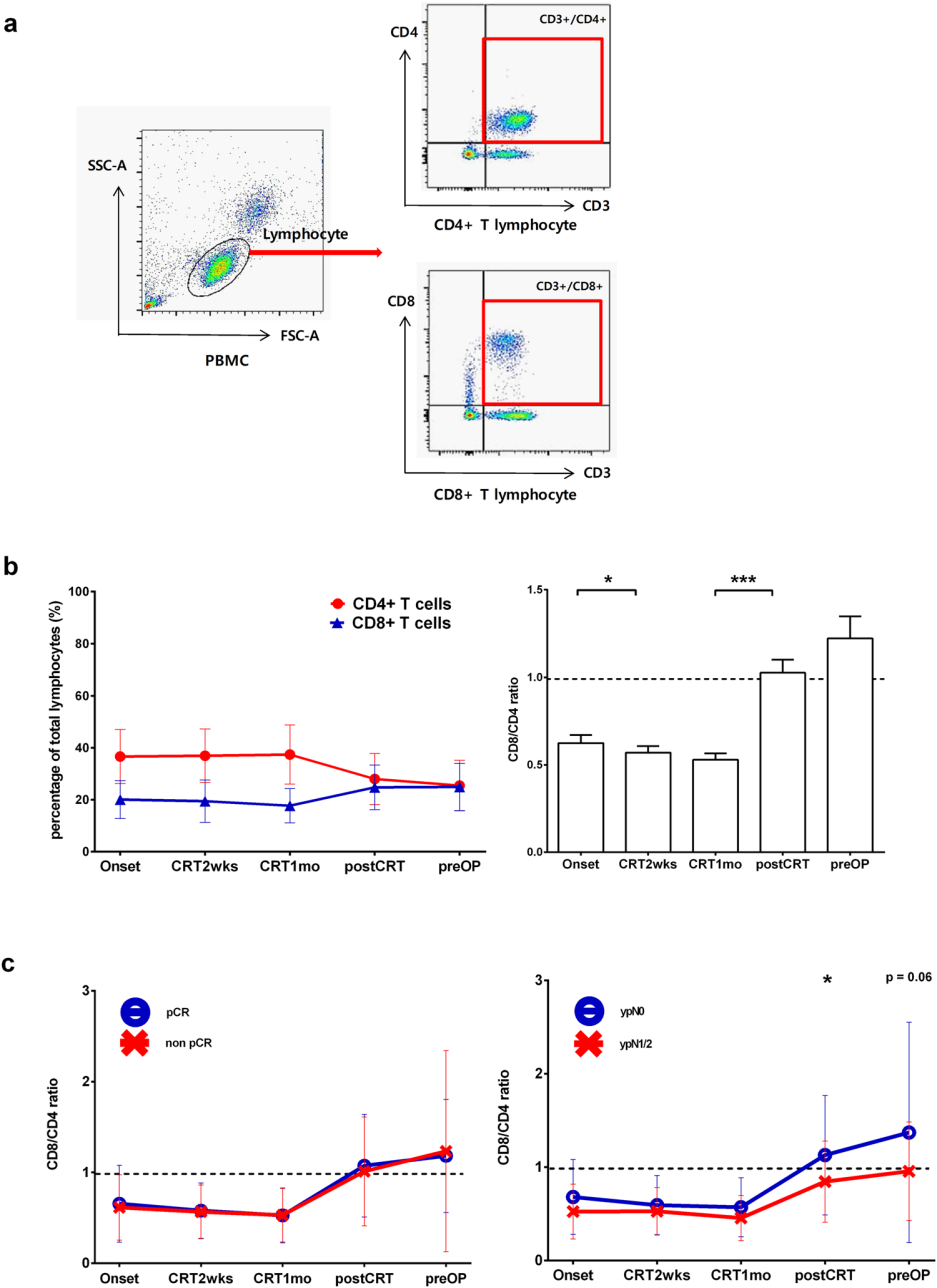
Changes in lymphocyte composition in response to CRT and the predictive value of these changes regarding pathological treatment response. (**a**) A flow cytometry plot shows the stratification of CD4+ T lymphocytes and CD8+ T lymphocytes. Lymphocytes were gated on a forward scatter by side scatter plot. Lymphocytes were subdivided into CD4+ T lymphocytes (CD3^+^CD4^+^) and CD8+ T lymphocytes (CD3^+^CD8^+^). (**b**) The proportions of CD8+ T lymphocytes and CD4+ T lymphocytes among total lymphocytes are presented in the left figure (n = 63). The ratio of CD8+ T lymphocytes to CD4+ T lymphocytes is presented in the right figure. Two-tailed paired Student’s t-test was used to analyse differences between two adjacent time points. *p < 0.05; **p < 0.0125 (adjusted significance level by Bonferroni’s method); ***p < 0.001. (**c**) The difference in the ratio of CD8+ T lymphocytes to CD4+ T lymphocytes between the pCR group (n = 15) and the non-pCR group (n = 49) is presented in the left figure. Repeated measures ANOVA; time effect p < 0.001; group effect p = 0.924; time-group interaction p = 0.838. The difference in the ratio of CD8+ T lymphocytes to CD4+ T lymphocytes between the ypN0 (no residual lymph node metastasis on pathological exam) group (n = 49) and the ypN1 or ypN2 (residual lymph node metastasis on pathological exam) group (n = 15) is presented in the right figure. Repeated measures ANOVA; time effect p < 0.001; group effect p = 0.07; time-group interaction p = 0.199. The intergroup differences were analysed using the two-tailed Welch’s t-test. *p < 0.05; **p < 0.01 (adjusted significance level by Bonferroni’s method); ***p < 0.001. Data are presented as the mean ± standard deviation. Onset = the onset of CRT; CRT2wks = 2 weeks after the onset of CRT; CRT1mo = 1 month after the onset of CRT; postCRT = 1 month after the termination of CRT; preOP = just before surgery.

Lymphocytes were further subdivided into CD4+ T lymphocyte cells (CD4+ T cells) and CD8+ T lymphocyte cells (CD8+ T cells) by flow cytometry analysis (Fig. 4a). The proportion of CD4+ T cells among total lymphocytes was relatively higher than that of CD8+ T cells during CRT, but after treatment was terminated, the proportion of CD8+ T cells increased and was similar to the proportion of CD4+ T cells (Fig. 4b). The ratio of CD8+ T cells to CD4+ T cells changed most dramatically from one month after CRT began to one month after CRT was terminated. The ratio of CD8 T+ cells to CD4+ T cells was not significantly associated with the pCR rate (Fig. 4c), but patients with residual lymph node metastasis had a lower ratio of CD8+ T cells to CD4+ T cells than patients without residual lymph node metastasis one month after the termination of CRT.

### Changes in peripheral cytokines and their predictive value

In the cytokine analysis, most INF-gamma values were below the range of the standard curve. Accordingly, we analysed the other five cytokines only. Although serum cytokine levels showed different patterns of change during and after CRT, all of the cytokines showed the most dramatic change from one month after CRT termination to just before surgery. Interleukin-12 (IL-12) levels decreased during CRT and began to recover one month after CRT was terminated. Monocyte chemotactic protein-1 (MCP-1) and macrophage inflammatory protein–1 alpha (MIP-1alpha) levels showed no significant change until one month after CRT was terminated, at which point they decreased significantly. Latency-associated peptide (LAP) and stromal cell-derived factor-1 alpha (SDF-1alpha) levels decreased during CRT, increased for one month after the termination of CRT, and decreased again thereafter. The serum levels of MCP-1 and MIP-1 alpha two weeks after the onset of CRT were significantly higher in patients without residual lymph node metastasis than in those with residual lymph node metastasis (Supplementary Figs S3, S4).

## Discussion

As more research shows that the antitumour immune response can be induced by chemotherapy and radiotherapy, there is increasing interest in whether CRT-mediated changes in the immune response can be accurately measured and used to predict therapeutic response^13^. In this context, we tracked the chronological changes in peripheral immune cell components, especially T lymphocytes and cytokines, elicited by CRT in patients with LARC. Subsequently, we assessed whether these parameters could predict the pathologic treatment response to CRT.

The absolute numbers of leukocytes and subtypes, including neutrophils, lymphocytes, and monocytes, decreased two weeks after the onset of CRT (Fig. 2a). This phenomenon may be explained by the impact of radiation therapy (RT) on pelvic bone marrow suppression or the haematologic toxicity of oral capecitabine^14,15^. Interestingly, during CRT, only the lymphocyte count continuously decreased. This finding is consistent with that of previous studies: lymphocytes are the most sensitive peripheral blood cell to RT^6,16,17^. The nadir monocyte and neutrophil counts occurred two weeks after the onset of CRT, and afterwards, these counts increased or were maintained. From these findings, we postulated that monocytes and neutrophils are potentially more resistant to CRT than lymphocytes and may be associated with treatment response. Indeed, we observed that treatment response was inversely related to monocyte and neutrophil counts. The counts of monocytes, neutrophils, and total leukocytes, but not of lymphocytes, were significantly lower in patients with a pCR than in those without a pCR two weeks after CRT initiation but not at CRT initiation (Fig. 3). The unfavourable role of monocytes and neutrophils in the pathologic tumour response might be associated with the well-known immunosuppressive role of tumour-associated macrophages (TAMs) and myeloid derived suppressor cells (MDSCs), but additional studies on this relationship are needed to reach firm conclusions^2^.

Numerous studies have aimed to discover reliable biomarkers predicting both long-term oncologic outcomes and the pathologic response to CRT in patients with LARC^5,6,18,19^. Previous studies showed that several parameters, such as the neutrophil to lymphocyte ratio (NLR), derived neutrophil to lymphocyte ratio (dNLR), lymphocyte percentage among leukocytes, and platelet count, could be associated with tumour response to CRT^6,17,20–22^. However, the conclusions from these previous studies have been discrepant. The cutoff values of NLR and the time points at which NLR was measured have varied among studies, which evaluated a limited number of time points both pre-CRT and post-CRT^6,21,22^. Additionally, the timing and frequency of blood sampling varied markedly among patients^17,20^. Notably, our study findings revealed that the differences in mean neutrophil and monocyte counts between patients with and without a pCR were pronounced two weeks after the onset of CRT. These findings might indicate that immune components change dynamically in response to CRT, and therefore, evaluating the immune response based on a single measurement would lead to flawed conclusions. Moreover, changes in neutrophil and monocyte counts during or after CRT, rather than at a single timepoint pre-CRT, could provide more valuable information for predicting pathologic tumour response.

The densities of CD8+ T cells and CD4+ T cells among tumour infiltrating lymphocytes (TILs) before CRT have been suggested to predict pathologic tumour response in rectal cancer^23,24^. Accordingly, we explored the changes in the proportions of CD8+ T cells and CD4+ T cells in peripheral blood. Interestingly, the proportion of CD8+ T cells, known as the core cell component in antitumour immunity, was maintained without any apparent change and was lower than that of CD4+ T cells during CRT (Fig. 4b). However, after the termination of CRT, the proportion of CD8+ T cells markedly increased to that of CD4+ T cells. Unlike TILs, the relative ratio of CD8+ T cells to CD4+ T cells was not correlated with pathologic response. Instead, the magnitudes of the increase in the proportion of CD8+ T cells and the decrease in the proportion of CD4+ T cells after CRT termination were significantly associated with residual lymph node metastasis (Fig. 4c). This finding may be related to the typical roles of the lymph node, in which naïve T lymphocytes first contact specific antigen, prompting T lymphocyte proliferation and differentiation into effector T lymphocytes. We postulate that the mere quantitative measurement of peripheral T cells might not be enough to predict treatment response and that specific characterization of peripheral T cells, which more accurately reflects TILs, might be required.

**Figure 5:**
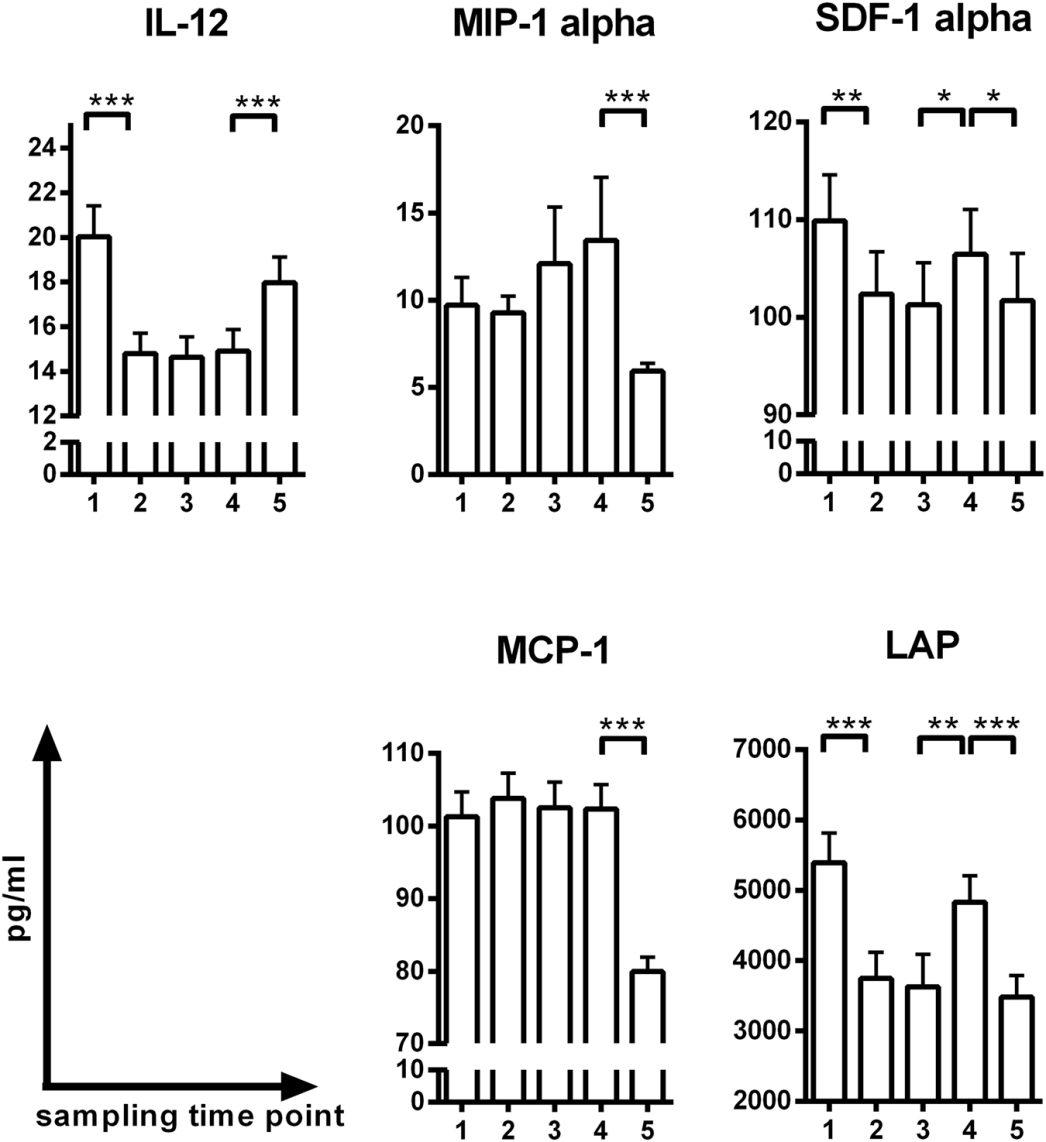
Changes in serum cytokine levels in response to CRT. Two-tailed paired Student’s t-test was used to analyse differences between two adjacent time points. *p < 0.05; **p < 0.0125 (adjusted significance level by Bonferroni’s method); ***p < 0.001. Data are presented as the mean ± standard deviation. 1 = the onset of CRT; 2 = 2 weeks after the onset of CRT; 3 = 1 month after the onset of CRT; 4 = 1 month after the termination of CRT; 5 = just before surgery.

To gain broad insight into the mechanisms that govern the systemic immune response, we investigated cytokines found to be related to chemoradiation or rectal cancer in previous studies^25–30^. The most striking finding of the analysed data is that dramatic changes in all cytokines commonly occurred from 1 month after CRT termination to just before surgery. IL-12 participates in antigen presentation and the licensing process between type I CD4+ helper T cells and antigen presenting cells (APCs) and is regarded as one of the main effectors of antitumour immunity. IL-12 levels increased dramatically one month after CRT termination (Fig. 5). In contrast to IL-12, the other four cytokines decreased during the same period. MCP-1, MIP-1alpha, and SDF-1 reportedly mediate resistance to radiotherapy or are involved in tumour progression in rectal cancer and other malignancies^25,26,28^. Additionally, TGF-beta, which was evaluated by measuring LAP levels in this study, is associated with poor oncologic outcomes in several cancers^27^. Therefore, we further identified the relationship between directional changes in cytokines and pathologic outcomes. While some cytokines measured at specific time points showed significant associations with pathologic tumour response, we could not clearly identify the predictive value of these relationships (Supplementary Figs S3, S4).

The current study has some limitations. We could not fully determine the diversity and heterogeneity of immune cells and cytokines to estimate and elucidate a precise immune response. Additionally, we only classified T lymphocytes as CD4+ T cells and CD8+ T cells. To better ascertain the role of lymphocytes, future research should use a more detailed classification of lymphocytes, including regulatory CD4+ T cells or cancer-specific CD8+ T cells, and functional stratification, such as the immune checkpoint expression or exhaustion profile (i.e., PD-1/CTLA-4 expression). Additionally, we should investigate more diverse cytokines concerning radiotherapy resistance or rectal cancer progression. Additionally, our study could not provide a complete explanation of the mechanism by which peripheral immune cells and cytokines are affected by the local immune response mediated by TILs. Lastly, we interpreted statistical significance as p < 0.05 in this study, even if multiple comparisons were performed; we noted the p-value adjusted by Bonferroni’s method. Thus, there might be an inflated type I error rate.

Our study is of importance in that we sequentially measured temporal changes in the immune response in patients receiving the same standard treatment, CRT, for a specific disease, LARC. We observed that peripheral immune components were affected by CRT and changed dramatically over time. Additionally, we identified biomarker candidates to predict the pathologic response in the future and provided insights into therapeutic strategies to improve CRT efficacy through immune modulation.

## Methods

### Patients

We prospectively enrolled 110 patients with locally advanced rectal cancer (LARC) who were scheduled to undergo preoperative chemoradiation therapy (CRT) and subsequent radical surgery and whose pathologic diagnosis was confirmed at Severance Hospital, Yonsei University College of Medicine between January 2015 and November 2016. In this study, we excluded patients who did not meet the following eligibility criteria: (1) rectal adenocarcinoma or mucinous carcinoma, and (2) prior completion of preoperative CRT followed by surgery with a curative aim. Finally, a total of 92 patients were included in this study, excluding 15 patients who withdrew study consent, 1 patient whose final pathology was confirmed as prostate cancer, and 2 patients who decided to undergo chemotherapy only after metastasis was proven through close evaluation (Supplementary Fig. S1).

### Clinicopathologic characteristics

Collected characteristics included age, sex, histopathological tumour characteristics, TNM stage, circumferential resection margin (CRM), primary tumour response to CRT, and residual lymph node stage (ypN). The 7th AJCC staging system was used to determine the TNM stage of the colorectal cancers. CRM involvement was evaluated by radiologists as invasion of the primary tumour into the proper mesorectal fascia. The primary tumour response to CRT was described by the tumour regression grading system suggested by Mandard *et al*.^31^.

### Preoperative CRT, surgery, and pathological examination

Indications for preoperative CRT included primary tumour stage higher than T3 or positive lymph node status based on clinical and radiological examinations. Preoperative CRT comprised oral capecitabine chemotherapy (825 mg/m^2^ twice daily for 3 weeks) and pelvic irradiation (45 Gy in 25 fractions administered to the entire pelvis, followed by a 5.4 Gy boost to the gross tumour administered in 3 fractions over 6 weeks or 50 Gy in 25 fractions administered to the entire pelvis over 5 weeks). Curative resection, including total or partial mesorectal excision depending on tumour characteristics, was performed within 12 weeks after CRT completion. Standardized pathological examination reports were completed for each case. The standard form required mandatory reporting of tumour differentiation, depth of tumour penetration, lymph node metastasis, CRM, lymphovascular invasion, and tumour regression grade.

### Blood sampling

All patients underwent peripheral venous blood sampling at five different time points: (1) the onset of CRT (just before CRT or on the CRT start day), (2) two weeks after the onset of CRT (cumulative radiation dose of approximately 18–19.8 Gy), (3) one month after the onset of CRT (cumulative radiation dose of approximately 45–46.8 Gy), (4) one month after CRT termination, and (5) just before surgery. All blood samples were obtained within 7 days of the scheduled time point.

### Complete blood count (CBC)

Peripheral venous blood samples, except for those obtained just before surgery, were analysed at the diagnostic test laboratory of our institution. White blood cell count, red blood cell count, platelet count and leukocyte differential count were calculated by automated cell counting machines or were manually counted by medical technologists using haemocytometers.

### Lymphocyte isolation, antibodies, immunophenotyping, and flow cytometry

Peripheral blood mononuclear cells (PBMCs) were isolated from whole blood by Ficoll-Paque (GE Healthcare, Uppsala, Sweden) density gradient centrifugation. Purified PBMCs were cryopreserved until use. For immunophenotyping, cryopreserved PBMCs were thawed and stained with the following fluorochrome-conjugated antibodies for 30 minutes at 4 °C and then washed once: anti-CD3 (clone: SK7, fluorochrome: APC-Cy7), anti-CD4 (RPA-T4, FITC), and anti-CD8 (RPA-T8, Percp-Cy5.5) (all from BD Biosciences). The cryopreserved PBMC samples from all time points for the same patient were thawed and analysed on the same day. Flow cytometry analyses were performed on an LSR II instrument with FACSDiva software (BD Biosciences). Subsequently, the data were analysed with FlowJo software (Treestar).

### Cytokine assay

Serum levels of interleukin-12 (IL-12), interferon gamma (INF-gamma), macrophage inflammatory protein-1 alpha (MIP-1alpha), monocyte chemotactic protein-1 (MCP-1), latency-associated peptide (LAP), and stromal cell-derived factor-1 alpha (SDF-1alpha) were determined by using customized ProcartaPlex multiplex immunoassays panels (Thermo Fisher Scientific, EPX060-19133-801) and a MAGPIX instrument (Thermo Fisher Scientific). Experiments were performed in duplicate, and the mean of values within the standard range is reported in this study.

### Statistical analyses

Categorical data are presented as the frequencies and percentages. Continuous data are presented as the means and standard deviations. The intergroup differences were analysed using the two-tailed Welch’s t-test. The assumption of a normal distribution was evaluated by the Kolmogorov-Smirnov normality test or by comparing a histogram of the sample data to a normal probability curve and checking whether the distribution graph had skewness < ±3 and kurtosis < ±7. Two-tailed paired Student’s t-test was used to compare matched samples. A p-value < 0.05 was considered significant, and confidence intervals (CIs) were calculated at the 95^th^ percentile level. GraphPad Prism ver. 5 (GraphPad Software, La Jolla, Ca) was used for statistical analysis and to present the analysed data as graphs.

### Ethics statement

All patients provided written informed consent after being given sufficient information on the study protocol. The study procedures were in accordance with the Declaration of Helsinki and were approved by the Institutional Review Board of Severance Hospital (IRB Number: 4-2014-0054).

### Data availability

The datasets generated and analysed in the current study are not publicly available due to patient privacy concerns but are available from the corresponding author on reasonable request.

## Electronic supplementary material

Supplementary information
