# Supplementary material for: Temporal changes in immune cell composition and cytokines in response to chemoradiation in rectal cancer
Source: Sci Rep. 2018 May 15;8:7565. doi: 10.1038/s41598-018-25970-z (PMC5953940; doi:10.1038/s41598-018-25970-z)
Supplement: Supplementary file 1 — Supplementary information [file 41598_2018_25970_MOESM1_ESM.pdf]

Temporal changes in immune cell composition and cytokines in response to chemoradiation in rectal cancer

Yong-Joon Lee[1], Sat-Byol Lee[2,3], Suk-Kyung Beak[2,3], Yoon-Dae Han[3], Min-Soo Cho[3], Hyuk Hur[3], Kang-Young Lee[3], Nam-Kyu Kim[3], Byung-Soh Min[2,3,*]

1 Graduate School of Medical Science and Engineering, Korea Advanced Institute of Science and Technology, Daejeon, Republic of Korea

2 Open NBI Convergence Technology Laboratory, Avison Biomedical Research Centre, Yonsei University College of Medicine, Seoul, Republic of Korea

3 Department of Surgery, Severance Hospital, Yonsei University College of Medicine, Seoul, Rebuplic of Korea

* Correspondence to :

Byung Soh Min, M.D. Department of Surgery, Severance Hospital, Yonsei University College of Medicine, 50-1 Yonsei-ro, Seodaemun-gu, 03722, Seoul, Republic of Korea; Tel: +82-2-2228-2100; Fax: +82-2-313-8289; E-mail: bsmin@yuhs.ac

Supplementary Figure S1

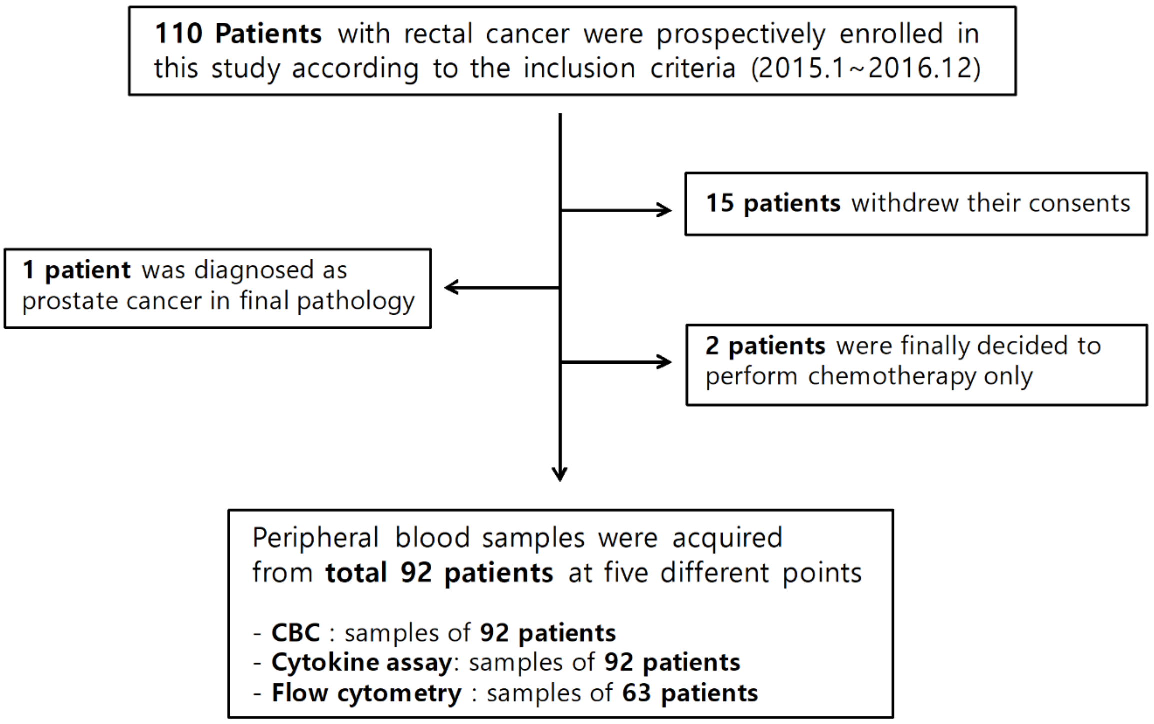

Supplementary Figure S1. Study flow diagram. CBC=complete blood count

Supplementary Figure S2

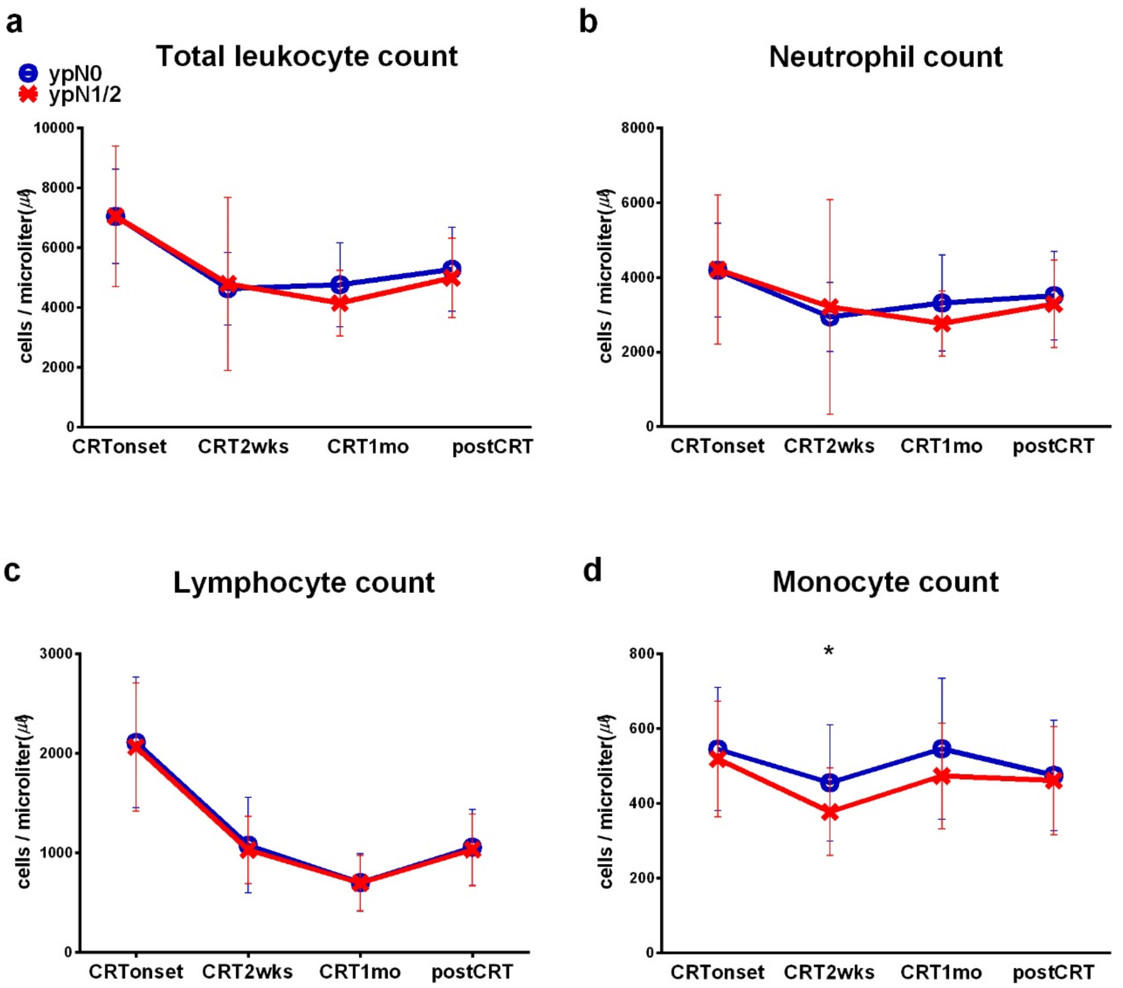

Supplementary Figure S2. Predictive value of leukocyte components regarding residual lymph node metastasis. Patients were divided into two groups: patients without a residual lymph node metastasis (ypN0, n=63) and patients with a residual lymph node metastasis (ypN1 or ypN2, n=29). a) Mean(±standard deviation, SD) total leukocyte counts of the two groups at each time point. Repeated-measures ANOVA; time effect p<0.001; group effect p=0.483; time-group interaction p=0.255. (b) Mean (±SD) neutrophil counts of the two groups at each time

point. Repeated-measures ANOVA; time effect $p<0.001$; group effect $p=0.020$; time-group interaction $p=0.120$. (c) Mean (±SD) lymphocyte counts of the two groups at each time point. Repeated-measures ANOVA; time effect $p<0.001$; group effect $p=0.583$; time-group interaction $p=0.939$. (d) Mean (±SD) monocyte counts of the two groups at each time point. Repeated-measures ANOVA; time effect $p<0.001$; group effect $p=0.067$; time-group interaction $p=0.258$. The intergroup differences were analysed using the two-tailed Welch's t-test. *$p<0.05$; **$p<0.0125$(adjusted significance level by Bonferroni's method); ***$p<0.001$. CRTonset=the onset of CRT; CRT2wks= 2 weeks after the onset of CRT; CRT1mo=1 month after the onset of CRT; postCRT=1 month after the termination of CRT.

Supplementary Figure S3

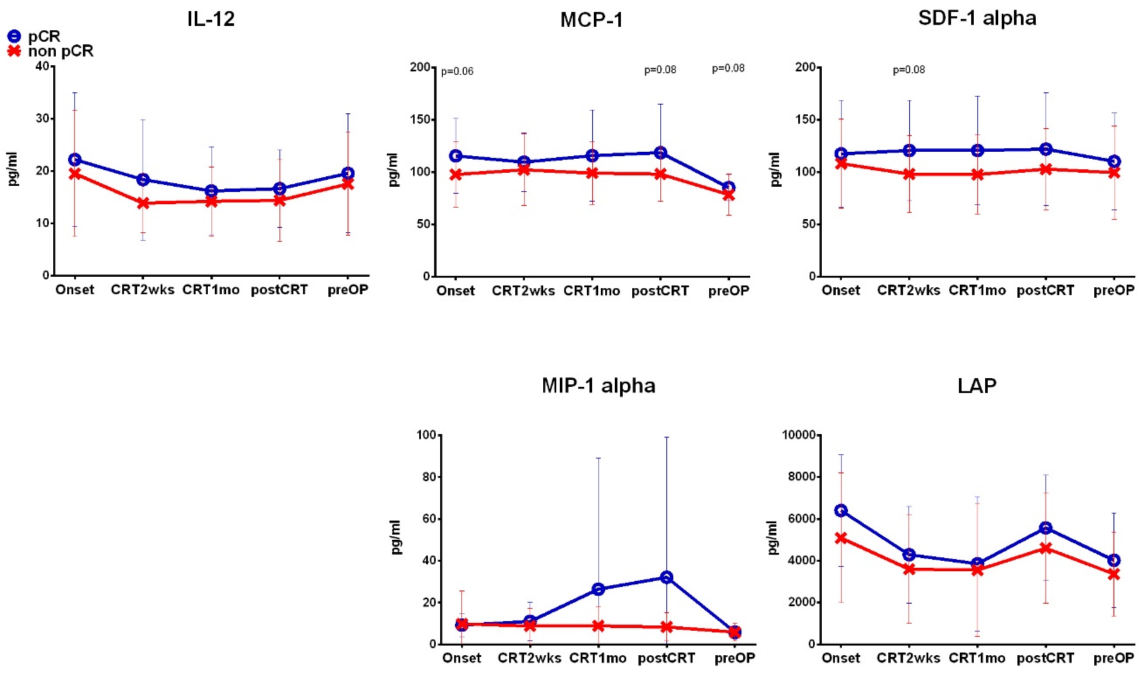

Supplementary Figure S3. Predictive value of serum cytokine levels regarding pathologic complete response (pCR). Patients were divided into two groups: patients with a pCR (n=19) and patients without a pCR (n=73). Two-tailed Welch's T-test was used. *p<0.05; **p<0.01 (adjusted significance level by Bonferroni's method); ***p<0.001. Data are presented as the mean±standard deviation. Onset=the onset of CRT; CRT2wks= 2 weeks after the onset of CRT; CRT1mo=1 month after the onset of CRT; postCRT=1 month after the termination of CRT; preOP=just before surgery.

Supplementary Figure S4

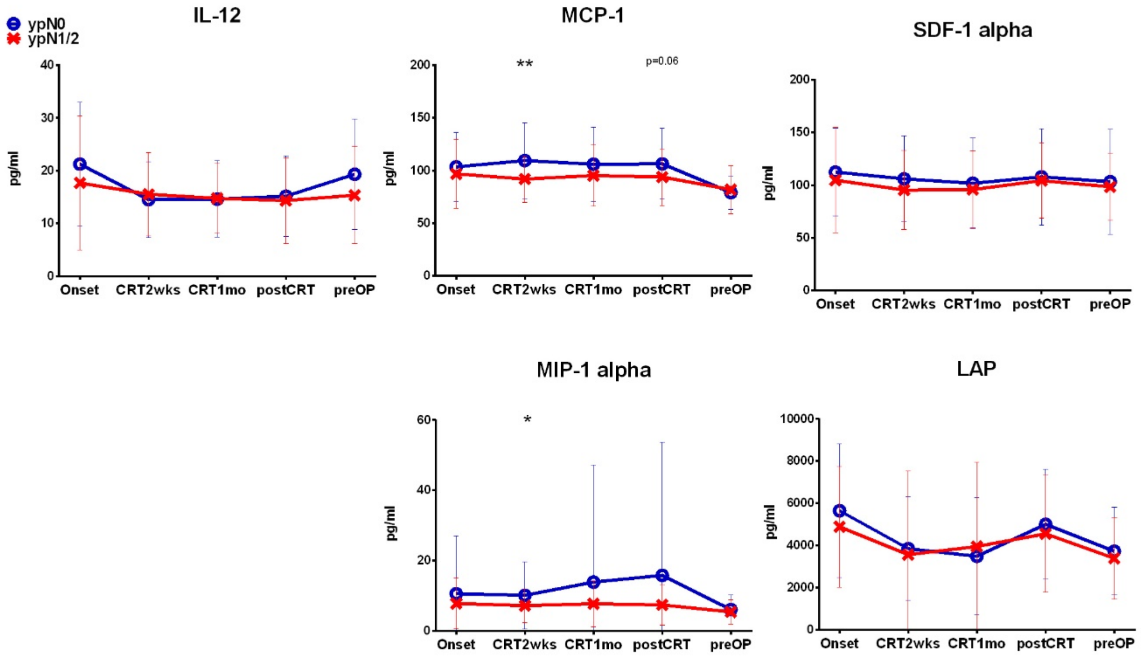

Supplementary Figure S4. Predictive value of serum cytokine levels regarding residual lymph node metastasis. Patients were divided into two groups: patients without a residual lymph node metastasis (ypN0, n=63) and patients with a residual lymph node metastasis (ypN1 or ypN2, n=29). Two-tailed Welch's T-test was used. *p<0.05; **p<0.01 (adjusted significance level by Bonferroni's method); ***p<0.001. Data are presented as the mean±standard deviation. Onset=the onset of CRT; CRT2wks= 2 weeks after the onset of CRT; CRT1mo=1 month after the onset of CRT; postCRT=1 month after the termination of CRT; preOP=just before surgery.
